# A Miniaturized Dual-Band Diplexer Design with High Port Isolation for UHF/SHF Applications Using a Neural Network Model

**DOI:** 10.3390/mi14040849

**Published:** 2023-04-14

**Authors:** Muhammad Akmal Chaudhary, Saeed Roshani, Salman Shabani

**Affiliations:** 1 Department of Electrical and Computer Engineering, College of Engineering and Information Technology, Ajman University, Ajman 346, United Arab Emirates; m.akmal@ajman.ac.ae; 2 Department of Electrical Engineering, Kermanshah Branch, Islamic Azad University, Kermanshah 6718997551, Iran

**Keywords:** bandpass filter, diplexer, dual band, resonator, interdigital filters, insertion loss

## Abstract

In this paper, a compact dual-band diplexer is proposed using two interdigital filters. The proposed microstrip diplexer correctly works at 2.1 GHz and 5.1 GHz. In the proposed diplexer, two fifth-order bandpass interdigital filters are designed to pass the desired frequency bands. Applied interdigital filters with simple structures pass the 2.1 GHz and 5.1 GHz frequencies and suppress other frequency bands with high attenuation levels. The dimensions of the interdigital filter are obtained using the artificial neural network (ANN) model, constructed from the EM-simulation data. The desired filter and diplexer parameters, such as operating frequency, bandwidth, and insertion loss, can be obtained using the proposed ANN model. The insertion loss parameter of the proposed diplexer is 0.4 dB, and more than 40 dB output port isolation is obtained (for both operating frequencies). The main circuit has the small size of 28.5 mm × 23 mm (0.32 λg × 0.26 λg). The proposed diplexer, with the achieved desired parameters, is a good candidate for UHF/SHF applications.

## 1. Introduction

The diplexer is an important element in RF transceivers which divides the input signal into two output ports, or vice versa [1]. In recent communication circuits and systems, simple structure, compact size, high-output port isolation, high rejection channel and low cost are required in diplexer design.

In many reported works, waveguide and cavity/dielectric resonators are used as popular methods to design high-isolation diplexers [2,3,4,5]. However, the heavy weight and large size of waveguide and cavity/dielectric components are not suitable for compact systems. Another popular method is the designing of two separate filters/resonators and combing them using T-junctions [6,7,8,9,10,11], which is suitable for compact diplexers design. Additionally, balanced diplexers with wide stop band can be easily obtained with the usage of two separate filters [8,9,10]. In [6,7,8,9,10,11], two independent filters and the T-junction combiners are located in the large circuit area.

Recently, neural network techniques have been used to improve the performance of electronic circuits, which also have been applied in the designing of the BPFs and diplexers [12,13,14,15,16,17]. Also, optical fibers substrates [18,19] can be used to operate at higher frequencies for filters and diplexers [20,21,22,23].

Moreover, to decrease the circuit size, in some reported works dual-band resonators are used in diplexers. For instance, a dual-mode stepped impedance resonator (SIR) is used in [24], but the output port isolation in this work is only 20 dB. In [25,26,27], diplexers and multiplexers are presented using T-shaped resonators. The output port isolation and insertion loss parameters are not good for these reported works.

To overcome these problems and achieve a small-size circuit, interdigital filters are used in some recently reported works. Interdigital filters with a simple structure are used for compact diplexer designs with a wide stop band in [28,29,30,31]. These filters include a series of coupled lines that are adjacent to each other. The mutual coupling occurs between adjacent lines, and the coupling of the non-adjacent line reaches zero. The widths of the resonators are typically the same; in this case, the filter is referred to as symmetric interdigital filters. The widths of the resonators can also be different, such as asymmetric interdigital filters. Although fixed-width resonators have a simple design, it is not always possible to design the desired circuit with a fixed-width resonator.

In [32], two dual-band filters (BPFs) are used to create a quad channel, but insertion losses at operating bands are high. In [32,33,34], neural network models are used to obtain the desired values of parameters in microwave devices, which resulted in optimum performances.

In this work, a high-isolation diplexer is designed with a compact size and two controllable frequencies. The proposed diplexer has low insertion loss and more than 14% and 4% fractional bandwidth for two operating frequency bands. Two separated fifth-order interdigital filters are used in two channels of the proposed diplexer, which has a simple structure, small size, and high port isolation.

The proposed diplexer is suitable for UHF/SHF applications. Ultra-high frequency (UHF) is the allocated frequency band for radio frequencies in the range between 300 MHz and 3 GHz, also known as the decimeter band. The first operating band of the proposed diplexer is located in this frequency range. The super-high frequency (SHF) is the allocated frequency band for radio frequencies (RF) in the range between 3 and 30 GHz, which is also known as the centimeter band. The second operating band of the proposed diplexer is located in this frequency range.

## 2. Design Process

**Figure 1:**
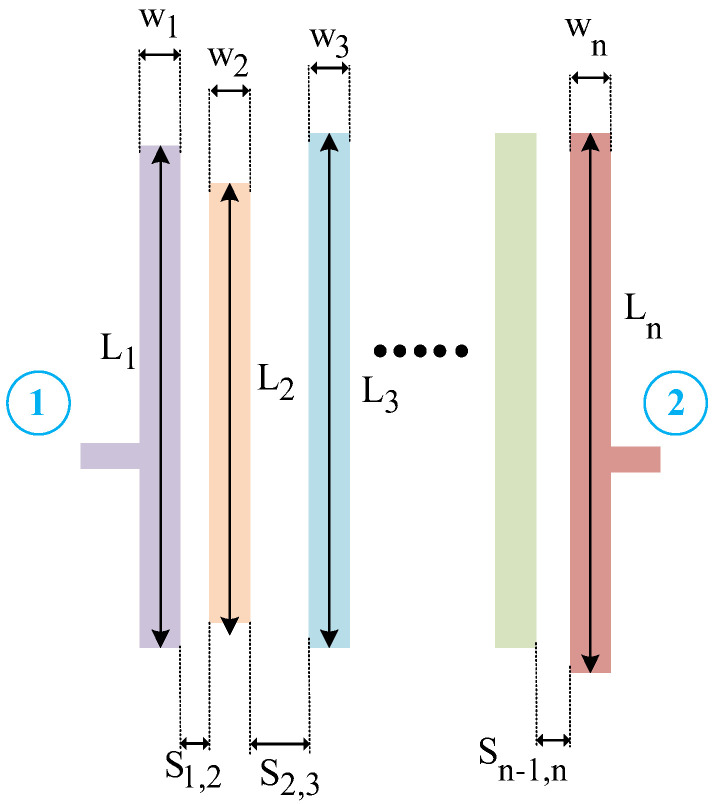
Structure of the typical interdigital filter.

The proposed diplexer consists of two interdigital BPFs. At the first BPF, the structure of the interdigital filter is investigated. A schematic of the typical interdigital BPF, which is widely used in microstrip applications [35], is illustrated in Figure 1. The filter configuration contains an array of n-lines.

In Figure 1, L_1_, L_2_, …, L_n_ indicate the length, and W_1_, W_2_, …, W_n_ show the width of the applied lines, respectively. The created fields between adjacent microstrip stubs cause the mutual coupling between these lines.

**Figure 2:**
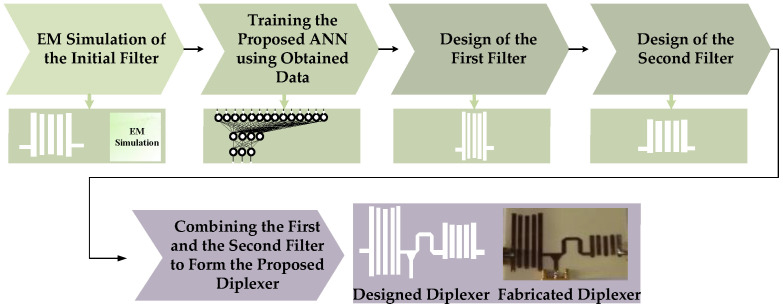
The design procedures of the proposed diplexer.

The design procedures of the proposed diplexer are shown in Figure 2. As seen, at first step, an initial filter is simulated several times with different dimensions and parameters to provide the desired data to train the neural network. Then, after training the neural network model, two filters are designed using the ANN predicted data, operating at the first and the second operating frequency bands. Then, the designed filters are combined to form the proposed diplexer with the desired parameters, which operates at the two desired main frequency bands.

**Figure 3:**
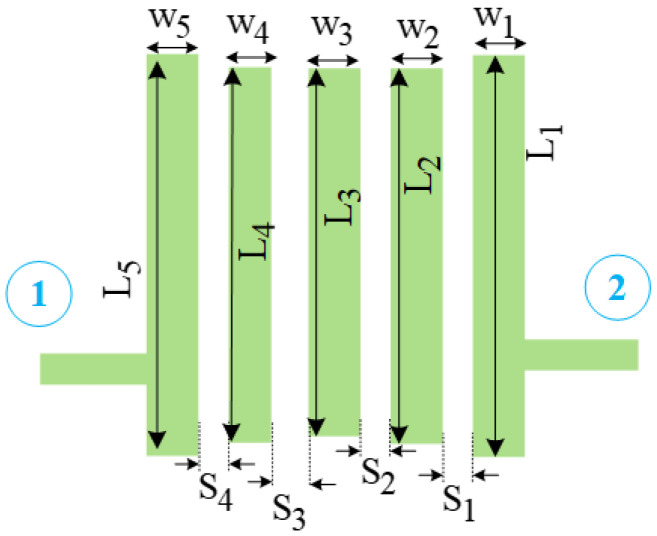
Structure of the general fifth-order interdigital filter.

In the proposed diplexer, two fifth-order interdigital filters are used in two channels. A schematic of the general fifth-order interdigital BPF is illustrated in Figure 3.

As seen in the applied fifth-order interdigital filter, 14 parameters are important to design the proposed filter. There are five microstrip lines, of which L_1_, L_2_, L_3_, L_4_, and L_5_ are the lengths of the five transmission lines, and W_1_, W_2_, W_3_, W_4_, and W_5_ are the widths of the five transmission lines. The parameters of S_1_, S_2_, S_3_, and S_4_ are the space gap between these five transmission lines. All these 14 parameters have important effects on the interdigital filter behavior, which determine the operating frequency, bandwidth, and insertion loss parameter of the filter. Hence, to obtain the desired values for the filters, the ANN model is proposed. In the proposed ANN model, these 14 mentioned parameters are used as input parameters of the neural network, and three parameters of operating frequency, bandwidth, and insertion loss are considered as the output parameters of the network.

## 3. The Architecture of the Proposed ANN Model

**Figure 4:**
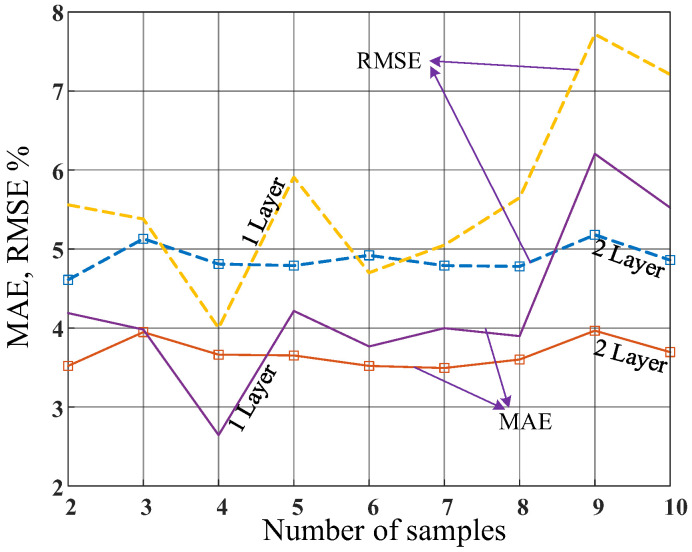
The obtained errors of the applied ANN models with different structures. The obtained errors are calculated by the 10 times running of each network with defined numbers of neurons and hidden layers. Also, the errors are calculated using normalized data, and the networks with 1 hidden layer and 2 hidden layers are applied.

A multilayer feed-forward neural network (ANN) is selected for the proposed model to predict the desired diplexer parameters, by considering the device dimensions. The feed-forward networks are also referred to as multilayer perceptrons (MLP), which are artificial neural networks that process data in a forward direction, from input to output, which do not revisit the same nodes again. The feed-forward ANNs consist of multiple layers of nodes, each with a set of biases and weights that are adjusted during the training phase to reduce the error between the network output and the target output. The input is fed to the first layer, which passes the output of that layer to the next layer, and so on, until the output is generated. The nonlinear activation function is applied to the input in each layer, which enables it to learn intricate patterns and features in the input data. The proposed model works as a surrogate model to predict parameters of the proposed interdigital filter. To obtained the best structure for the ANN model, several models with different layers are examined. The obtained errors of the applied ANN models with different structures are shown in Figure 4. As can be seen, the ANN model with only one hidden layer with 4 neurons in the hidden layer is the best structure, which also has low complexity.

**Figure 5:**
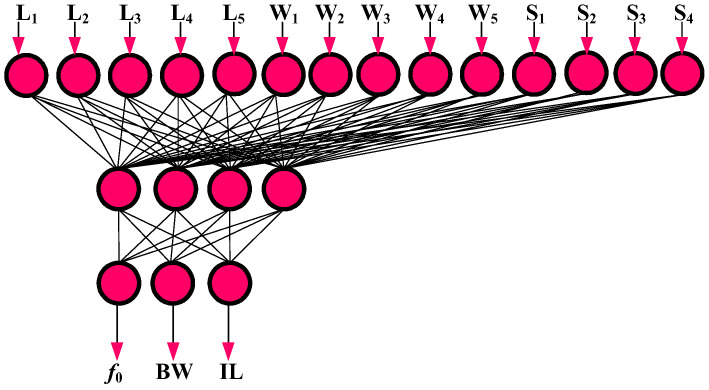
Proposed structure of the ANN model including a single hidden layer with four neurons in the hidden layer.

Figure 5 shows the proposed MLP structure for the defined artificial network. According to this figure, the input parameters are connected to the output nodes by a single hidden layer including four neurons in the hidden layer. After finding the best structure, the proposed ANN model is tested 100 times with the epochs of 200 up to 1000, to obtain the best values of the proposed ANN model.

In the presented ANN model, 31 samples are used for the training of the network, while 7 and 2 samples are used for the test and validation of the proposed surrogate model, respectively. In the proposed ANN, mean relative error (MRE) and root mean square error (RMSE) are considered to evaluate the proposed model results as follows.

(1) $$MRE=\frac{1}{N}\sum_{i=1}^{N}\left|\;\frac{Y_{Ri}-Y_{Pi}}{Y_{Ri}}\right|$$

(2) $$RMSE=\sqrt{\frac{\sum_{i=1}^{N}{\left(Y_{Ri}-Y_{Pi}\right)}^{2}}{N}}$$

where *N* is total number of the dataset, *Y_Ri_* and *Y_Pi_* are the real and predicted output of the presented ANNs, respectively.

### Results of the Proposed ANN Model

**Figure 6:**
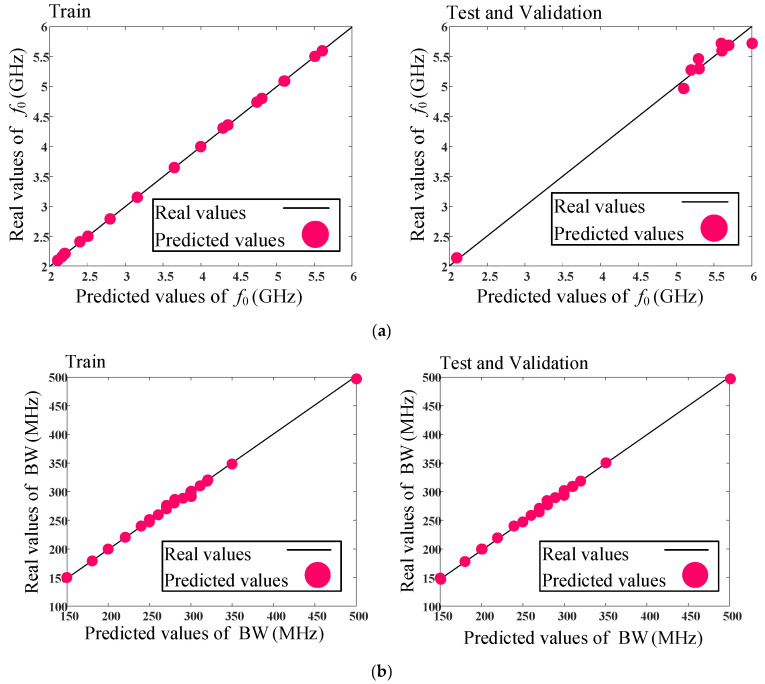

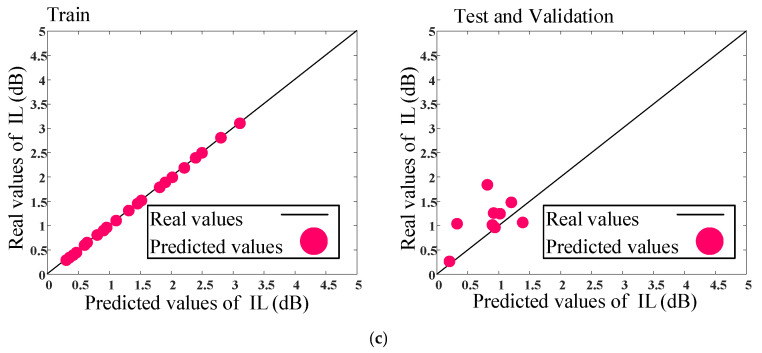
The real and predicted comparison values for train and test data, using the proposed model. Real and predicted values of (**a**) *f*_0_ (GHz), (**b**) BW (MHz), and (**c**) IL (dB).

As mentioned, the presented model with structure of a single hidden layer and four neurons in the hidden layer is selected as the most precise model. The real and predicted comparison values of *f*_0_ (GHz), BW (MHz), and IL (dB) parameters for train and test data are shown in Figure 6. As can be seen, the predicted data are obtained accurately.

**Figure 7:**
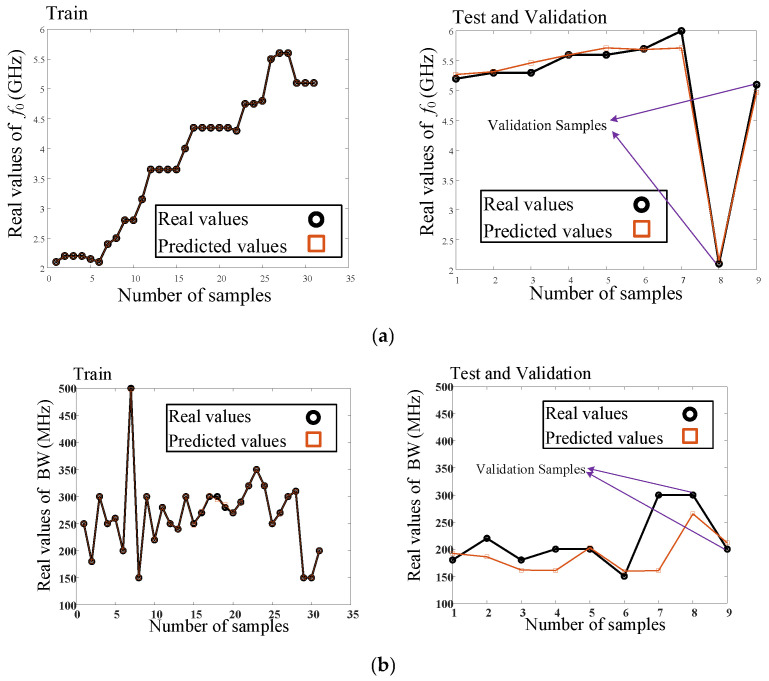

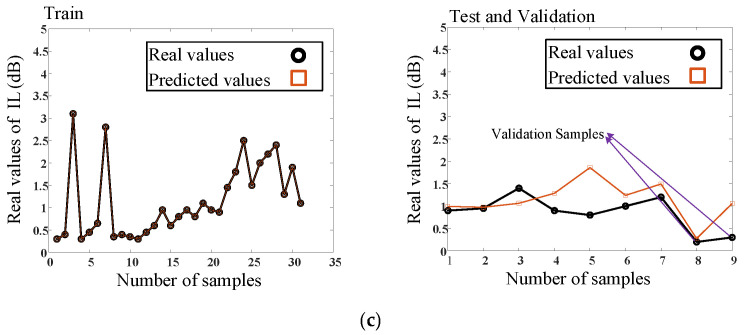
Real and predicted test and train values of (**a**) *f*_0_ (GHz), (**b**) BW (MHz), and (**c**) IL (dB) parameters versus number of data samples in the proposed model.

Real and predicted test and train values of *f*_0_ (GHz), BW (MHz), and IL (dB) parameters versus the number of data samples in the proposed model are shown in Figure 7. As seen, the circuit parameters are predicted accurately. Two samples are chosen for validation, which are shown in Figure 7. The validation samples validate the accuracy of the proposed model and help to design the proposed interdigital filter with the desired parameters.

The real data of train, test, and validation procedures, using the proposed model, are listed in Table 1. The final results of the proposed ANN model are listed in Table 2, which show high accuracy of the prediction for the proposed model. The errors which are reported in this table correspond to the denormalized data. According to this table, the model is trained perfectly using the train data.

## 4. Circuit Configuration

**Figure 8:**
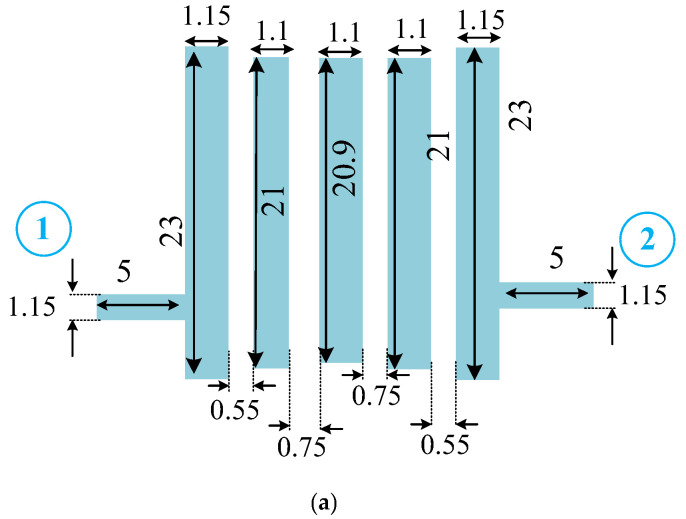

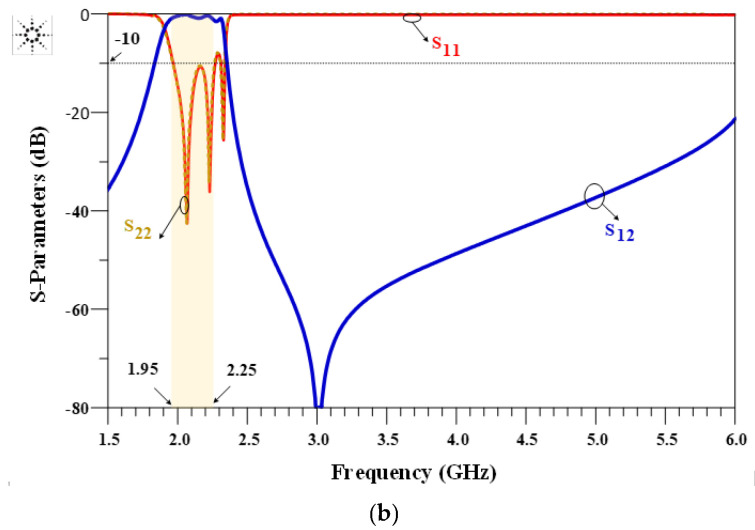
Designed 2.1 GHz filter. (**a**) Circuit structure, and (**b**) S-parameters simulated frequency response (dimensions are in mm).

**Figure 9:**
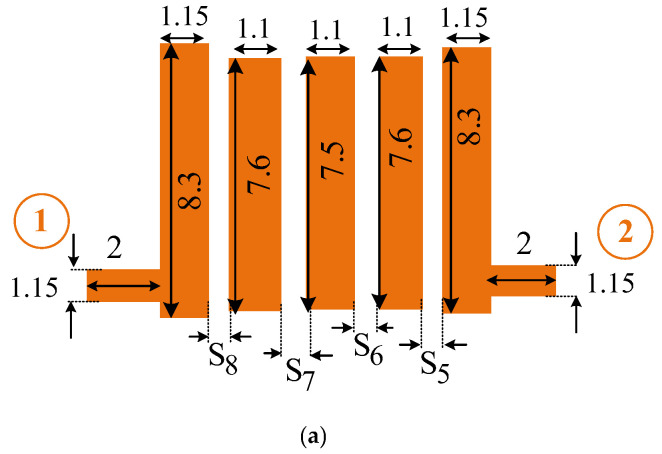

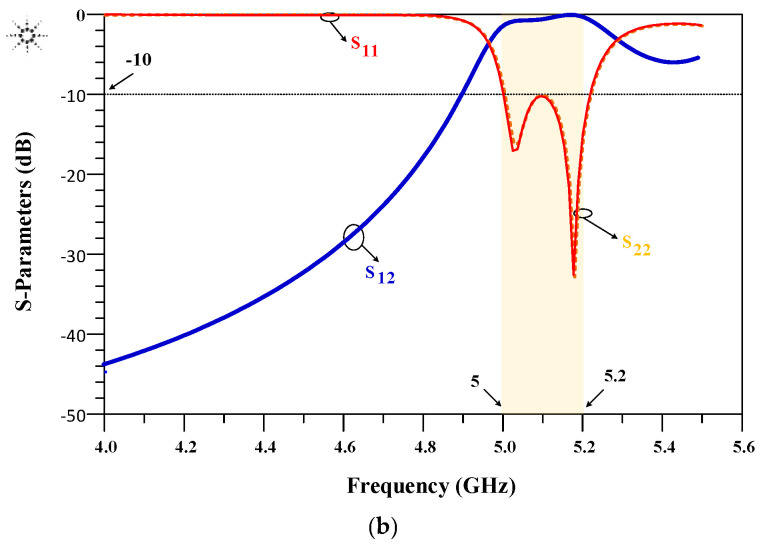
Designed 5.1 GHz filter. (**a**) Circuit structure, and (**b**) S-parameters simulated frequency response (dimensions are in mm).

To investigate the performance of two designed filters, a standard Rogers 4003 substrate with 20-mil thickness, 3.38 dielectric constant, and loss tangent of 0.0022 is used. The simulations are done using ADS software. The final circuit configuration and frequency responses of these two designed filters are demonstrated in Figure 8 and Figure 9. The dimensions of the depicted proposed BPF in Figure 8 are obtained from the ANN model and extracted from the first row of the validation values from Table 1.

The proposed BPF correctly works at 2.1 GHz with 0.3 dB insertion loss. The proposed BPF has good operating bandwidth of 300 MHz from 1.95 to 2.25 GHz, which shows 14.3% FBW. Due to the reciprocal and symmetrical structure of the proposed BPF, S_11_ and S_22_ are similar.

The dimensions of the depicted proposed BPF in Figure 9 are obtained from the ANN model and extracted from the second row of the validation values from Table 1.

The proposed BPF correctly works at 5.1 GHz with 0.4 dB insertion loss. The proposed BPF has good operating bandwidth of 200 MHz from 5 to 5.2 GHz, which shows 4% FBW. Due to the reciprocal and symmetrical structure of the proposed BPF, S_11_ and S_22_ are similar.

## 5. Diplexer Configuration

**Figure 10:**
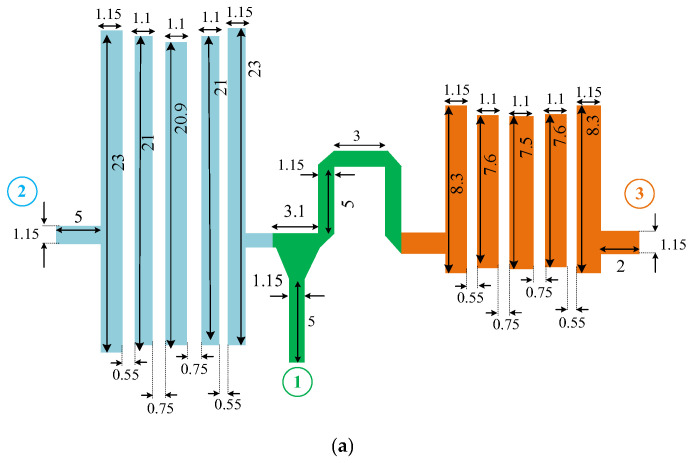

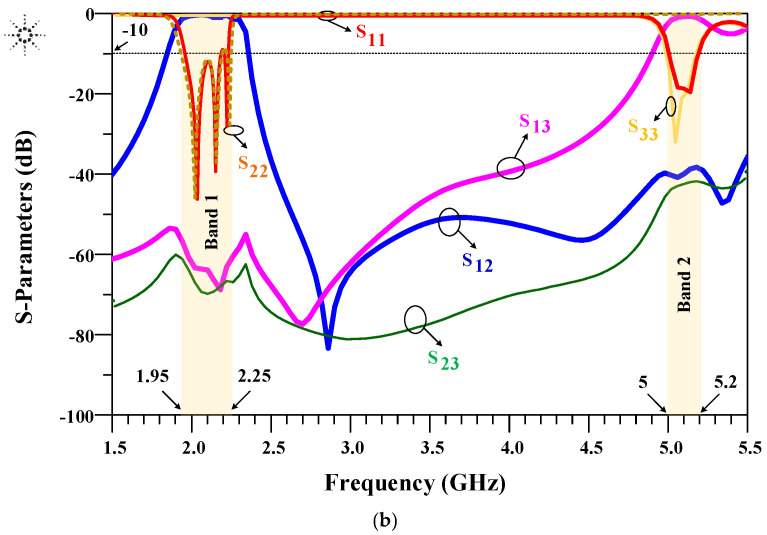
Designed diplexer. (**a**) Circuit structure, and (**b**) S-parameters simulated frequency response (dimensions are in mm).

As mentioned in the previous section, in order to form a diplexer, two independent filters must be combined with a T-junction. The location of these two proposed filters and the T-junction are very effective at the return loss, the insertion loss, and the matching parameters. The final circuit configuration of the proposed diplexer and its frequency response are depicted in Figure 10.

One of the main challenges in the design of a diplexer is the output port isolation (S_23_). As shown in Figure 10b, the proposed design has high isolation between output ports at the two pass bands. The isolation levels between two output ports (S_23_) through the two pass bands are better than 40 dB, which confirm the validity of the analysis. Due to the reciprocal and symmetrical structure of the proposed BPFs, the S_11_ and S_22_ are similar at the first operating band, while S_11_ and S_33_ are similar at the second operating band.

**Figure 11:**
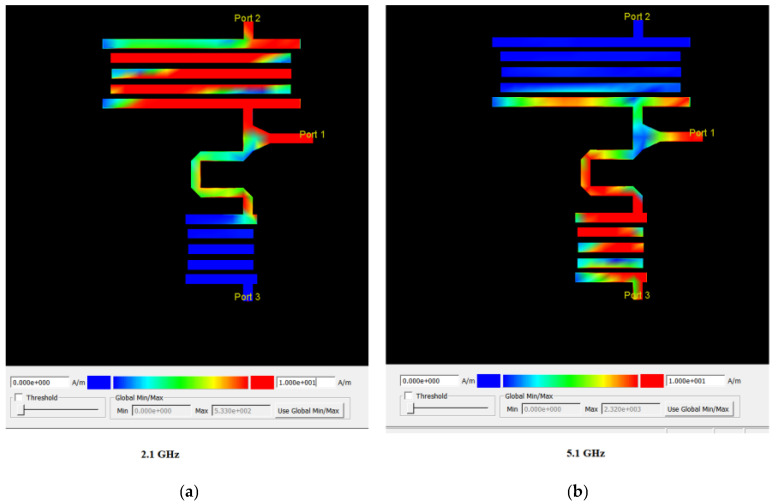
Surface current distribution in the proposed diplexer at the frequencies of (**a**) 2.1 GHz, first operating frequency band in port 2, (**b**) 5.1 GHz, second operating frequency band in port 3.

The surface current distribution in the proposed diplexer is demonstrated in Figure 11. The proposed diplexer correctly works at two frequency bands of 2.1 and 5.1 GHz. As the results show in Figure 11a, the currents are correctly distributed uniformly at port 2 at 2.1 GHz frequency, which shows that the currents have not reached port 3.

Also, in Figure 11b, the results show that the currents are correctly distributed uniformly at port 3 at 5.1 GHz frequency, which shows that the currents have not reached port 2.

## 6. Implementation and Experimental Results

**Figure 12:**
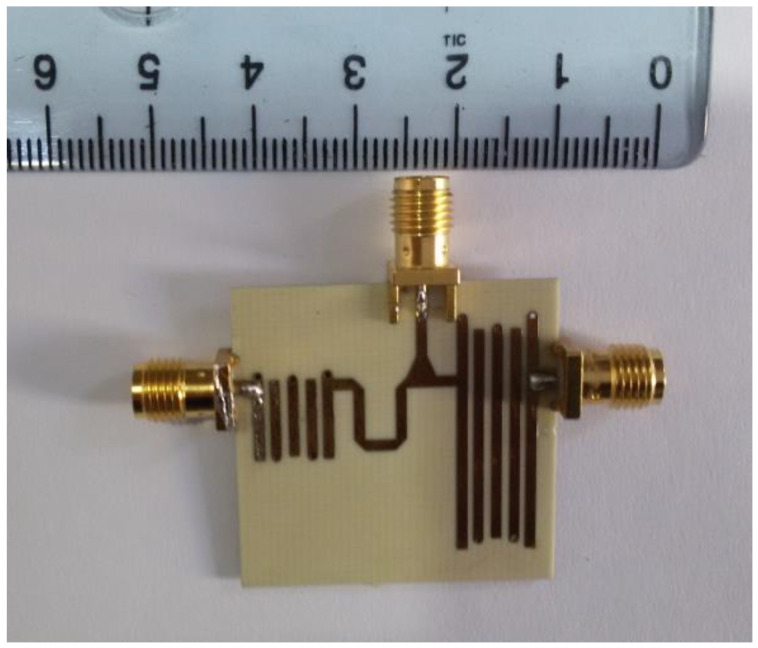
Fabricated photo of the proposed microstrip diplexer with two fifth-order interdigital BPFs.

The designed diplexer was implemented, and the photo of the fabricated diplexer prototype is illustrated in Figure 12. The overall size of the proposed circuit is 28.5 mm × 23 mm (0.32 λg × 0.26 λg).

**Figure 13:**
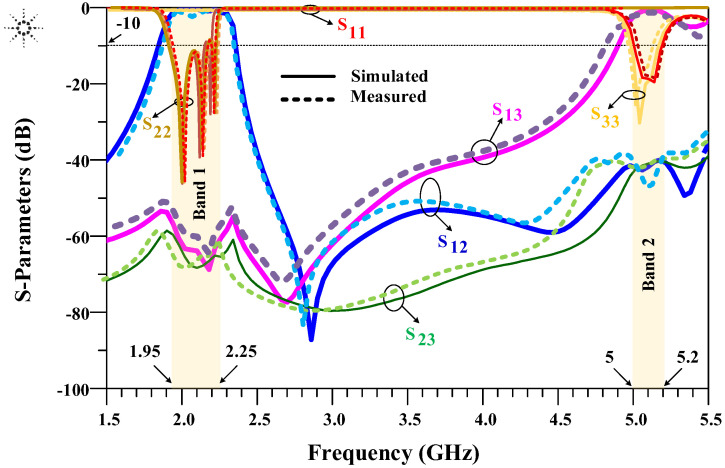
Simulations and measurements of S-parameters results of the fabricated diplexer.

The proposed device using interdigital filters is fabricated and tested by a keysight technologies 8720B Network Analyzer. The simulations results and the measurements results of S-parameters are depicted in Figure 13. The results indicate that the minimum insertion loss of the proposed diplexer is less than 0.4 dB, and the return loss is higher than 40 dB. As demonstrated in Figure 13, a good agreement between the simulation and the measurement results is achieved. Little mismatch between the simulation results and measurements results could be explained to the fabrication tolerance or/and the change of the material properties.

Table 3 summarizes the performances of the designed diplexer and some similar references. The diplexer performances in terms of the insertion loss, output port isolation, structure complexity, circuit size, mid-band frequency, and fractional bandwidth are listed in Table 3. The proposed device has a simple structure and has an easy fabrication process; moreover, it has a small size due to implementation of the interdigital filters.

## 7. Conclusions

This paper proposed two interdigital bandpass filters operating at 2.1 GHz and 5.1 GHz, designed by the proposed ANN model. Based on these two proposed interdigital filters, a compact size diplexer is proposed for the UHF/SHF band. The desired dimensions of these two applied interdigital bandpass filters are obtained using a neural network model. The proposed microstrip diplexer has a small size, high port isolation between two channels, and a low insertion loss. The measurement results of the diplexer show excellent agreement with the ADS simulation results. The results show that the insertion loss is about 0.4 dB in both operating frequencies, and the output port isolation is more than 40 dB, which are desirable parameters.

## Figures and Tables

**Table 1:** The real data of train, test, and verification procedures, using the proposed model.

L_1_ (mm)		L_2_ (mm)	L_3_ (mm)	L_4_ (mm)	L_5_ (mm)	W_1_ (mm)	W_2_ (mm)	W_3_ (mm)	W_4_ (mm)	W_5_ (mm)	S_1_ (mm)	S_2_ (mm)	S_3_ (mm)	S_4_ (mm)	F_o_ (GHz)	BW (MHz)	IL (dB)
Train Values of Design Parameters																	
**Input Parameters**															**Output Parameters**		
1	22	21	20.9	21	22	1.15	1.1	1.1	1.1	1.15	0.55	0.75	0.75	0.55	2.1	250	0.30
2	22	20	19	20	22	1.15	1.1	1.1	1.1	1.15	0.55	0.75	0.75	0.55	2.2	180	0.40
3	22	20	21	20	22	1.15	1.1	1.1	1.1	1.15	0.55	0.75	0.75	0.55	2.2	300	3.10
4	22	20	19	20	22	1.15	1.1	1.3	1.1	1.15	0.55	0.65	0.65	0.55	2.2	250	0.30
5	22	20	19	20	22	1.15	1.3	1.3	1.3	1.15	0.45	0.55	0.55	0.45	2.15	260	0.45
6	20	20	19	20	20	1.15	1.3	1.3	1.3	1.15	0.45	0.55	0.55	0.45	2.1	200	0.65
7	20	18	19	18	20	1.15	1.3	1.3	1.3	1.15	0.45	0.55	0.55	0.45	2.4	500	2.80
8	20	18	17	18	20	1.4	1.3	1.3	1.3	1.4	0.30	0.55	0.55	0.30	2.5	150	0.35
9	18	16	15	16	18	1.4	1.3	1.3	1.3	1.4	0.30	0.55	0.55	0.30	2.8	300	0.40
10	18	16	15	16	18	1.4	1.1	1.3	1.1	1.4	0.40	0.65	0.65	0.40	2.8	220	0.35
11	16	14	13	14	16	1.4	1.1	1.3	1.1	1.4	0.40	0.65	0.65	0.40	3.15	280	0.30
12	14	12	11	12	14	1.4	1.1	1.3	1.1	1.4	0.40	0.65	0.65	0.40	3.65	250	0.45
13	14	12	11	12	14	1.3	1.1	1.3	1.1	1.3	0.45	0.65	0.65	0.45	3.65	240	0.60
14	14	12	11	12	14	1.2	1.1	1.3	1.1	1.2	0.50	0.65	0.65	0.50	3.65	300	0.95
15	14	12	11	12	14	1.2	1.1	1.1	1.1	1.2	0.50	0.75	0.75	0.50	3.65	250	0.60
16	13	11	10	11	13	1.2	1.1	1.1	1.1	1.2	0.50	0.75	0.75	0.50	4.0	270	0.80
17	12	10	9	10	12	1.2	1.1	1.1	1.1	1.2	0.50	0.75	0.75	0.50	4.35	300	0.95
18	12	10	9	10	12	1.2	1.1	1.0	1.1	1.2	0.50	0.80	0.80	0.50	4.35	300	0.80
19	12	10	9	10	12	1.1	1.1	1.0	1.1	1.1	0.55	0.80	0.80	0.55	4.35	280	1.10
20	12	10	9	10	12	1.1	1.2	1.0	1.2	1.1	0.50	0.75	0.75	0.50	4.35	270	0.95
21	12	10	9	10	12	1.1	1.3	1.0	1.3	1.1	0.45	0.70	0.70	0.45	4.35	290	0.90
22	12	10	9	10	12	1.1	1.3	1.2	1.3	1.1	0.45	0.60	0.60	0.45	4.30	320	1.45
23	11	9	8	9	11	1.1	1.3	1.2	1.3	1.1	0.45	0.60	0.60	0.45	4.75	350	1.80
24	11	9	8	9	11	1.1	1.1	1.2	1.1	1.1	0.55	0.70	0.70	0.55	4.75	320	2.50
25	11	9	8	9	11	1.2	1.1	1.1	1.1	1.2	0.50	0.75	0.75	0.50	4.80	250	1.50
26	10	8	7	8	10	1.2	1.1	1.1	1.1	1.2	0.50	0.75	0.75	0.50	5.5	270	2.0
27	9.5	7.5	6.5	7.5	9.5	1.2	1.1	1.1	1.1	1.2	0.50	0.75	0.75	0.50	5.6	300	2.2
28	9.0	7.5	6.5	7.5	9.0	1.2	1.1	1.1	1.1	1.2	0.50	0.75	0.75	0.50	5.6	310	2.4
29	8.5	7.5	8	7.5	8.5	1.2	1.1	1.1	1.1	1.2	0.50	0.75	0.75	0.50	5.1	150	1.3
30	8.0	7.5	7.5	7.5	8.0	1.2	1.1	1.1	1.1	1.2	0.50	0.75	0.75	0.50	5.1	150	1.9
31	8.0	7.5	7.5	7.5	8.0	1.15	1.1	1.1	1.1	1.15	0.55	0.75	0.75	0.55	5.1	200	1.1
	**Test Values**																
1	8.3	7.0	7.5	7.0	8.3	1.15	1.1	1.1	1.1	1.15	0.55	0.75	0.75	0.55	5.2	180	0.9
2	8.0	7.0	7.5	7.0	8.0	1.15	1.1	1.1	1.1	1.15	0.55	0.75	0.75	0.55	5.3	220	0.95
3	8.0	7.0	7.0	7.0	8.0	1.15	1.1	1.1	1.1	1.15	0.55	0.75	0.75	0.55	5.3	180	1.4
4	7.5	7.0	7.0	7.0	7.5	1.15	1.1	1.1	1.1	1.15	0.55	0.75	0.75	0.55	5.6	200	0.9
5	7.5	7.0	6.5	7.0	7.5	1.15	1.1	1.1	1.1	1.15	0.55	0.75	0.75	0.55	5.6	200	0.8
6	7.5	6.5	6.5	6.5	7.5	1.15	1.1	1.1	1.1	1.15	0.55	0.75	0.75	0.55	5.7	150	1
7	7.0	6.5	6.5	6.5	7.0	1.15	1.1	1.1	1.1	1.15	0.55	0.75	0.75	0.55	6	300	1.2
	**Validation Values**																
1	23	21	20.9	21	23	1.15	1.1	1.1	1.1	1.15	0.55	0.75	0.75	0.55	2.1	300	0.20
2	8.3	7.6	7.5	7.6	8.3	1.15	1.1	1.1	1.1	1.15	0.55	0.75	0.75	0.55	5.1	200	0.30

**Table 2:** The results of the proposed ANN model.

	*f*_o_ (GHz) Errors			BW (MHz) Errors			IL (dB) Errors		
	Train	Test	Valid	Train	Test	Valid	Train	Test	Valid
MRE	1.48 × 10^−6^	0.0169	0.0260	0.0024	0.1520	0.0867	6.54 × 10^−7^	0.3734	1.4483
RMSE	1.17 × 10^−5^	0.1349	0.0994	1.5628	57.0586	25.8761	9.01 × 10^−6^	0.4697	0.5385

**Table 3:** Comparison between the proposed diplexer and some related designs.

	Structure	Isolation (dB)	Dimensions λg × λg	*f*_o_ (GHz)	Insertion Loss (dB)	Return Loss (dB)	FBW (%)
[36]	Complex	51	0.12 × 0.11	1.075/1.71	2.7/2.9	-	2
[37]	Complex	62.5	69.08 × 41.49	1.95/2.14	0.968/0.942	20	-
[38]	Simple	40	-	0.9/1.8	0.4/0.5	18	9
[39]	Complex	70	-	60.5/65.5	1.1/1.37	-	50
[40]	Complex	40	-	1.9/3.4	5/5.4	10	5
[41]	Simple	41	0.29 × 0.23	1.8/2.2	1.25/1.55	5	5
[42]	Complex	21	-	1.8/2.45	2.2/2.2	16	4
[43]	Complex	20	-	2/3	1/1	20	6
[44]	Complex	60	0.14 × 0.12	0.5/0.86	2.75/2.9	14	6
This work	Simple	60/40	0.32 × 0.26	2.1/5.1	0.3/0.4	10/18	14/4

## Data Availability

All the material conducted in the study is mentioned in the article.
